# The association between the dietary index for gut microbiota and frailty: a cross-sectional study

**DOI:** 10.3389/fnut.2025.1580753

**Published:** 2025-05-01

**Authors:** Shouxin Wei, Sijia Yu, Meng Sun

**Affiliations:** ^1^Department of Gastrointestinal Surgery, Suining Central Hospital, Suining, China; ^2^Department of General Practice, Suining Central Hospital, Suining, China

**Keywords:** diet index, gut microbiota, dietary index for gut microbiota, frailty, NHANES

## Abstract

**Background:**

Frailty severely impacts patients’ quality of life and imposes a significant burden on healthcare systems. The Dietary Index for Gut Microbiota (DI-GM) is an emerging dietary indicator, and its association with frailty has not been thoroughly investigated.

**Methods:**

This study utilized data from NHANES 2007–2018 and assessed the association between DI-GM and frailty using multivariable weighted logistic regression, restricted cubic splines (RCS), subgroup analysis, and mediation analysis, after adjusting for relevant covariates.

**Results:**

The results indicate a significant negative correlation between DI-GM and frailty, with each standard unit increase in DI-GM reducing the risk of frailty by 6% (OR = 0.940 [0.899, 0.984]). DI-GM at different quartiles showed a strong dose–response relationship, with the highest quartile showing a 21.6% risk reduction. RCS analysis revealed a linear relationship between DI-GM and frailty. Subgroup analysis suggested that age and smoking status may influence the association between DI-GM and frailty. Furthermore, albumin and high-density lipoprotein (HDL) played significant mediating roles in the relationship between DI-GM and frailty, accounting for 30.34 and 9.05% of the total effect, respectively.

**Conclusion:**

Dietary Index for Gut Microbiota is negatively associated with frailty risk, and albumin and HDL mediate this association. Improving dietary quality may be an effective strategy for reducing frailty risk.

## 1 Introduction

Currently, the world is facing an increasingly severe aging problem. Meanwhile, the incidence of frailty is also rising year by year (1, 2). Frailty is a complex syndrome marked by diminished physical function, decreased mobility, and inadequate physiological reserves, resulting in various health issues, including falls, hospitalization, dementia, and heightened mortality risk (3). Frailty greatly impacts patients’ quality of life and imposes a heavy financial strain on healthcare systems (4). Studies have found that frailty can not only be prevented but also mitigated through effective interventions (5). Therefore, understanding and identifying the factors associated with frailty is crucial for improving the health of older adults.

The gut microbiota plays a key role in maintaining human health and function, and recent studies have shown that it is closely linked to the onset and progression of frailty. As people age, the gut microbiota’s diversity and stability typically diminish, with overgrowth of harmful bacteria and a reduction in beneficial bacteria. This imbalance is considered one of the potential factors contributing to frailty (6). The “gut-brain axis” significantly contributes to frailty onset, as alterations in gut microbiota may affect cerebrovascular health and cognitive function in older adults via neural and hormonal pathways, potentially worsening frailty symptoms (7). Moreover, the gut microbiota engages with the immune system, metabolic processes, and general health (8, 9).

Diet plays a crucial role in shaping the composition and function of the gut microbiota, and specific dietary components have been shown to exert profound effects on its composition and function. Dietary fiber is one of the primary substrates for gut microbial fermentation, leading to the production of short-chain fatty acids (SCFAs), which regulate the immune system, maintain gut barrier integrity, and suppress inflammatory responses (10). Prebiotics and probiotics promote the growth of beneficial bacteria and enhance microbial ecosystem stability (11). In addition, polyphenolic compounds such as tea polyphenols, quercetin, and flavonoids contribute to host health by modulating specific microbial taxa and metabolic pathways (12). The “diet–microbiota–inflammation axis” may be involved in the pathogenesis of frailty, as demonstrated by Ghosh et al. (13) whose study showed that adherence to the Mediterranean diet modulated the gut microbiota composition in older adults, reduced inflammation, and subsequently mitigated frailty progression and improved overall health. Building upon this, Kase et al. (14) performed a literature review and examined current longitudinal research on diet and gut microbiota, identifying 14 foods associated with gut microbiota and developing the Dietary Index for Gut Microbiota (DI-GM). By integrating multiple dietary factors, DI-GM assesses the influence of diet on gut microbiota, providing a standardized tool for evaluating the dietary influence on gut microbiota. Diet, as an economic and efficient intervention strategy, plays a vital role in promoting gut health. Albumin and high-density lipoprotein (HDL) may play a critical role in the relationship between diet, gut microbiota, and frailty. Malnutrition often leads to decreased serum albumin levels, thereby accelerating the progression of frailty (15). HDL possesses antioxidant and anti-inflammatory properties and may influence the development and progression of frailty by modulating systemic inflammation and metabolic status (16). However, no studies have yet explored the association between DI-GM and frailty.

This study aims to explore the association between DI-GM and frailty by analyzing NHANES data from 2007 to 2018. Based on existing evidence that healthy dietary patterns—such as the Mediterranean diet and diets rich in dietary fiber and polyphenols—improve gut microbiota and reduce frailty risk, we hypothesized that higher DI-GM scores would be inversely associated with a lower risk of frailty. The findings of this study may not only enhance our understanding of the relationship between diet and frailty, but also provide a scientific basis for developing targeted nutritional interventions for older adults, and serve as a critical reference for future public health nutrition policies and geriatric care guidelines.

## 2 Methods

### 2.1 Study population

**Figure 1:**
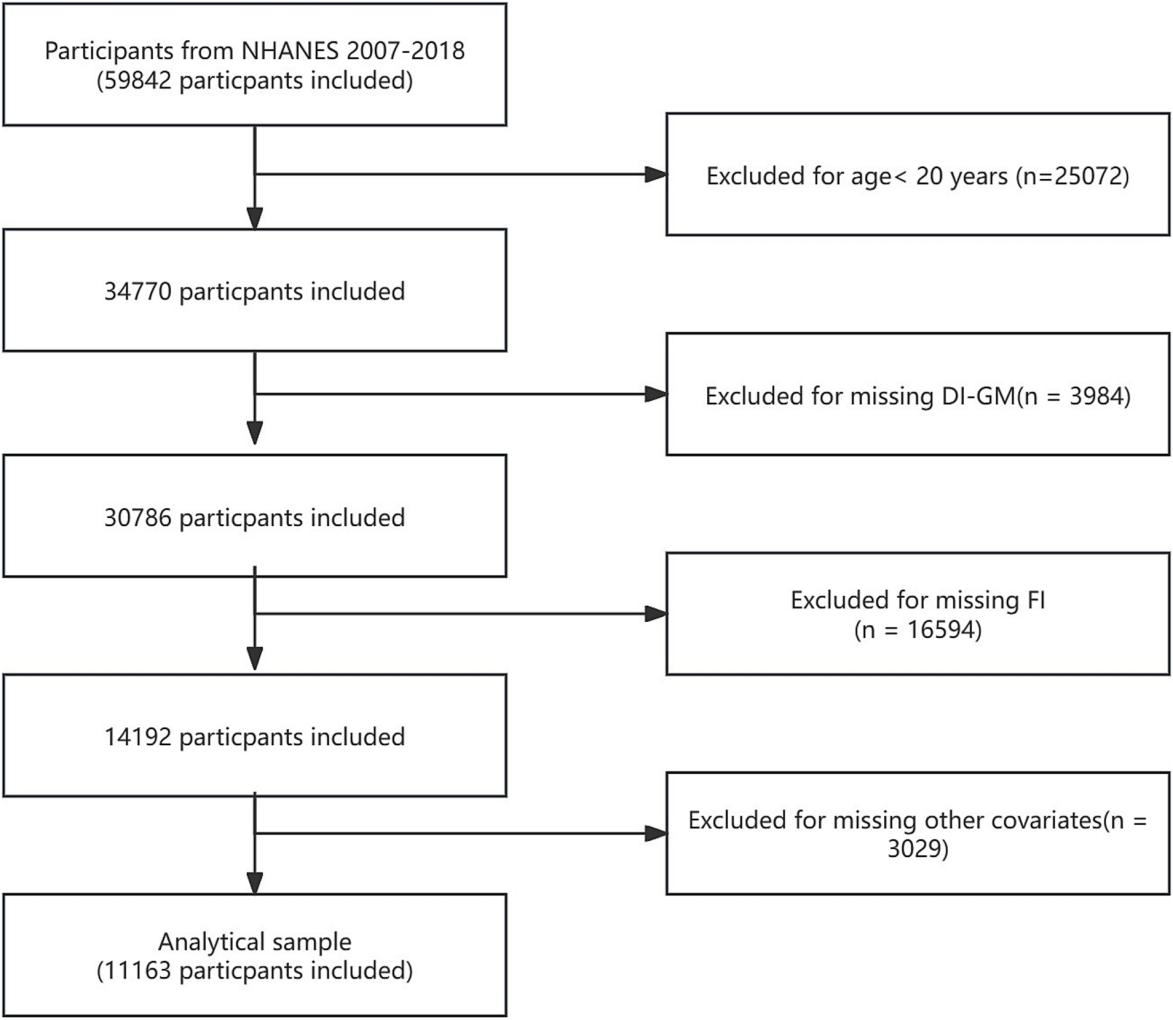
Flowchart of the study.

NHANES is a significant national health survey project sponsored by the Centers for Disease Control and Prevention (CDC). The survey not only covers individuals’ physiological health status but also thoroughly analyzes health trends related to socioeconomic background, dietary habits, and environmental factors through detailed questionnaires and physical examinations. The entire data collection process strictly adheres to ethical requirements, ensuring the privacy and rights of all participants are fully protected. For more information on NHANES ethical review and consent procedures, please visit NHANES Ethical Review. The data for this study was sourced from 59,842 participants in NHANES 2007–2018. Exclusion criteria included: (1) participants aged < 20 years (*n* = 25,072); (2) participants missing DI-GM data (*n* = 3,984); (3) participants with incomplete or poor frailty index (FI) data (*n* = 16,594); and (4) participants lacking other covariate data (*n* = 3,029). In the end, 11,163 individuals were part of the final analysis. Figure 1 provides a detailed overview of participant selection.

### 2.2 Exposure assessment

This research utilized the scoring method suggested by Kase et al. (14), which assesses the DI-GM index by calculating the intake of 10 beneficial foods for gut health and 4 potentially harmful foods. The index is based on NHANES 24-h dietary recall data and scores participants based on whether their intake reaches the gender-specific median. For beneficial foods, participants who consume above the median score 1, otherwise they score 0; for harmful foods, participants who consume below the median score 1, otherwise they score 0. The DI-GM total score ranges from 0 to 14, with a higher score generally indicating better gut microbiota health. Participants were grouped into four categories according to their DI-GM total scores: 0–3, 4, 5, and 6 or greater (17, 18). The relevant calculation criteria are presented in Supplementary Table S1.

### 2.3 Outcome assessment

The FI is calculated based on the cumulative deficit model, which is defined by evaluating 49 items across various health domains (19). In this study, inclusion criteria required participants to complete at least 39 items, covering physical measurements, chronic comorbidities, healthcare utilization, physical dependence, laboratory tests, and depressive symptoms. Items were rated on a scale from 0 to 1, with 1 signifying the greatest severity of the associated deficit. The FI score is determined by dividing the number of existing deficits by the total number of items, yielding a value from 0 to 1, where 0 means no deficits and 1 signifies the highest deficit burden. Based on previous studies, the FI cutoff value was set at 0.25, with individuals exceeding this value classified as frail and those below this value considered non-frail (20, 21). All scoring criteria and details are provided in Supplementary Table S2.

### 2.4 Covariates

We included several relevant covariates in the analytical model. Sociodemographic factors included age, gender, race, educational background, marital status, and poverty-to-income ratio (PIR). Lifestyle factors included smoking and alcohol consumption. The classification of marital status included three categories: married or cohabitating, divorced/separated/widowed, and never married. Education level was categorized based on years of schooling into three levels: high school or less, high school graduate, and post-secondary education. Income levels for PIR were divided into low (<1.3), middle (1.3–3.49), and high (≥3.5). Smoking status was classified based on history and current smoking behavior: former smokers were individuals who had stopped smoking but had consumed over 100 cigarettes in their lifetime; current smokers were those who were actively smoking and had smoked 100 or more cigarettes. Those who had never smoked or had smoked under 100 cigarettes were considered non-smokers. Alcohol consumption was classified similarly, non-drinkers are defined as individuals who have had less than 12 alcoholic beverages in their entire life. Former drinkers are individuals who have had 12 or more drinks but consumed less than one drink in the last year and current drinkers are defined as those who have had 12 or more drinks and at least one drink in the last year.

### 2.5 Statistical analysis

NHANES employs a complex multi-stage probability sampling method to ensure the sample is broadly representative. Following the NHANES analysis manual, we applied sampling weights and accounted for primary sampling units and stratification factors in the data analysis. Our study included participants from six cycles, thus the laboratory weight of 1/6 was applied in the data analysis. Continuous variables are displayed as mean ± SD, and categorical variables are expressed as percentages *t*-tests, Wilcoxon rank-sum tests, and chi-square tests were used to assess the significance of intergroup differences. The association between DI-GM and frailty was investigated using weighted logistic regression. Model 1 was unadjusted, Model 2 controlled for factors such as age, gender, and race, and Model 3 adjusted for age, gender, race, education level, marital status, PIR, smoking, and alcohol consumption. Results are displayed using weighted odds ratios (OR) and 95% confidence intervals (95% CI). We employed restricted cubic spline regression models to investigate the dose–response relationship between DI-GM and frailty, aiming to identify possible threshold points. Stratified analysis and interaction tests were conducted to verify the robustness of the results. Additionally, mediation analysis was conducted using the R package “mediation,” with 1,000 bootstrap resampling iterations to evaluate the mediating roles of albumin and HDL in the association between DI-GM and frailty. The direct effect reflected the impact of DI-GM on frailty, while the indirect effect quantified the mediating roles of albumin and HDL. The proportion of the mediation effect was calculated as: indirect effect/(indirect effect + direct effect) × 100%. All statistical analyses were performed using R software (version 4.4.1) and EmpowerStats (version 4.2), and a *p*-value of less than 0.05 was considered statistically significant.

## 3 Results

### 3.1 Participant characteristics

The study comprised 11,163 participants, with an average age of 62.33 ± 14.43 years, including 5,520 males and 5,643 females. Among all participants, 2,901 were diagnosed with frailty, and 8,262 were non-frail individuals. Table 1 indicates that, relative to the non-frail group, the frail group consisted of older participants with a higher percentage of females and non-Hispanic Black individuals. They also had lower educational attainment, a greater proportion of divorced/separated/widowed individuals, lower PIR, and higher percentages of former drinkers and current smokers. Additionally, the frail group exhibited lower levels of albumin and HDL.

**Table 1:** Baseline characteristics of the frailty and non-frailty groups in the 2007–2018 NHANES.

Characteristics	Total (*n* = 11,163)	Non-frailty (*n* = 8,262)	Frailty (*n* = 2,901)	*P*-value
Age (years)	62.33 ± 14.43	62.15 ± 14.74	62.85 ± 13.48	0.025
Sex				<0.001
Male	5,520 (49.45%)	4,288 (51.90%)	1,232 (42.47%)	
Female	5,643 (50.55%)	3,974 (48.10%)	1,669 (57.53%)	
Race				<0.001
Mexican American	1,306 (11.70%)	972 (11.76%)	334 (11.51%)	
Non-Hispanic Black	2,244 (20.10%)	1,587 (19.21%)	657 (22.65%)	
Non-Hispanic White	5,658 (50.69%)	4,223 (51.11%)	1,435 (49.47%)	
Other Hispanic	1,099 (9.85%)	811 (9.82%)	288 (9.93%)	
Other Race	856 (7.67%)	669 (8.10%)	187 (6.45%)	
Education level				<0.001
Below high school	3,099 (27.76%)	2013 (24.36%)	1,086 (37.44%)	
High school	2,735 (24.50%)	2030 (24.57%)	705 (24.30%)	
Above high school	5,329 (47.74%)	4,219 (51.07%)	1,110 (38.26%)	
Marital status				<0.001
Married/Living with partner	6,335 (56.75%)	4,934 (59.72%)	1,401 (48.29%)	
Divorced, separated, or widowed	3,670 (32.88%)	2,451 (29.67%)	1,219 (42.02%)	
Never married	1,158 (10.37%)	877 (10.61%)	281 (9.69%)	
PIR				<0.001
<1.3	3,895 (34.89%)	2,462 (29.80%)	1,433 (49.40%)	
1.3–3.49	4,316 (38.66%)	3,216 (38.93%)	1,100 (37.92%)	
≥3.5	2,952 (26.44%)	2,584 (31.28%)	368 (12.69%)	
Drinking status				<0.001
Never	1758 (15.75%)	1,265 (15.31%)	493 (16.99%)	
Former	2,554 (22.88%)	1,633 (19.77%)	921 (31.75%)	
Now	6,851 (61.37%)	5,364 (64.92%)	1,487 (51.26%)	
Smoking status				<0.001
Never	5,203 (46.61%)	4,064 (49.19%)	1,139 (39.26%)	
Former	3,777 (33.83%)	2,760 (33.41%)	1,017 (35.06%)	
Now	2,183 (19.56%)	1,438 (17.40%)	745 (25.68%)	
Albumin	4.17 ± 0.33	4.21 ± 0.31	4.05 ± 0.36	<0.001
HDL	53.15 ± 16.58	54.15 ± 16.61	50.30 ± 16.17	<0.001

PIR, poverty-to-income ratio; HDL, high-density lipoprotein; n indicates the sample size of the study.

### 3.2 Association between DI-GM and frailty

The results from the multivariable weighted logistic regression analysis are shown in Table 2. In Model 3, treating DI-GM as a continuous variable revealed that each standard unit increase corresponded to a 6% decrease in frailty risk (OR = 0.940 [0.899, 0.984]). When DI-GM was treated as a categorical variable, a strong dose–response relationship was observed with frailty. As DI-GM increased, the negative association with frailty became more pronounced. Compared to the group with a DI-GM score of 0–3, the risk of frailty in the ≥6 group was reduced by 21.6% (OR = 0.784 [0.653, 0.941]).

**Table 2:** Multivariable linear regression association between DI-GM and frailty.

Exposure	Model 1 OR (95% CI)	Model 2 OR (95% CI)	Model 3 OR (95% CI)
Continuous DI_GM	0.903 (0.866, 0.941) < 0.001	0.899 (0.861, 0.939) < 0.001	0.940 (0.899, 0.984) 0.01
DI-GM group			
0–3	1[Reference]	1[Reference]	1[Reference]
4	1.000 (0.842, 1.188) 0.999	0.997 (0.836, 1.189) 0.974	1.024 (0.853, 1.230) 0.797
5	0.777 (0.658, 0.917) 0.004	0.776 (0.655, 0.920) 0.005	0.856 (0.723, 1.013) 0.074
≥6	0.669 (0.565, 0.792) < 0.001	0.659 (0.552, 0.787) < 0.001	0.784 (0.653, 0.941) 0.011
P for trend	<0.001	<0.001	0.002

OR, odds ratio; 95% CI, 95% confidence interval. DI-GM, dietary index for gut microbiota; Model 1: no adjustment for covariates. Model 2: adjusted for age, gender, and race. Model 3: adjusted for age, gender, race, education level, marital status, PIR, smoking, alcohol consumption. *p* < 0.05 is considered statistically significant.

### 3.3 Dose–response analysis between DI-GM and frailty

**Figure 2:**
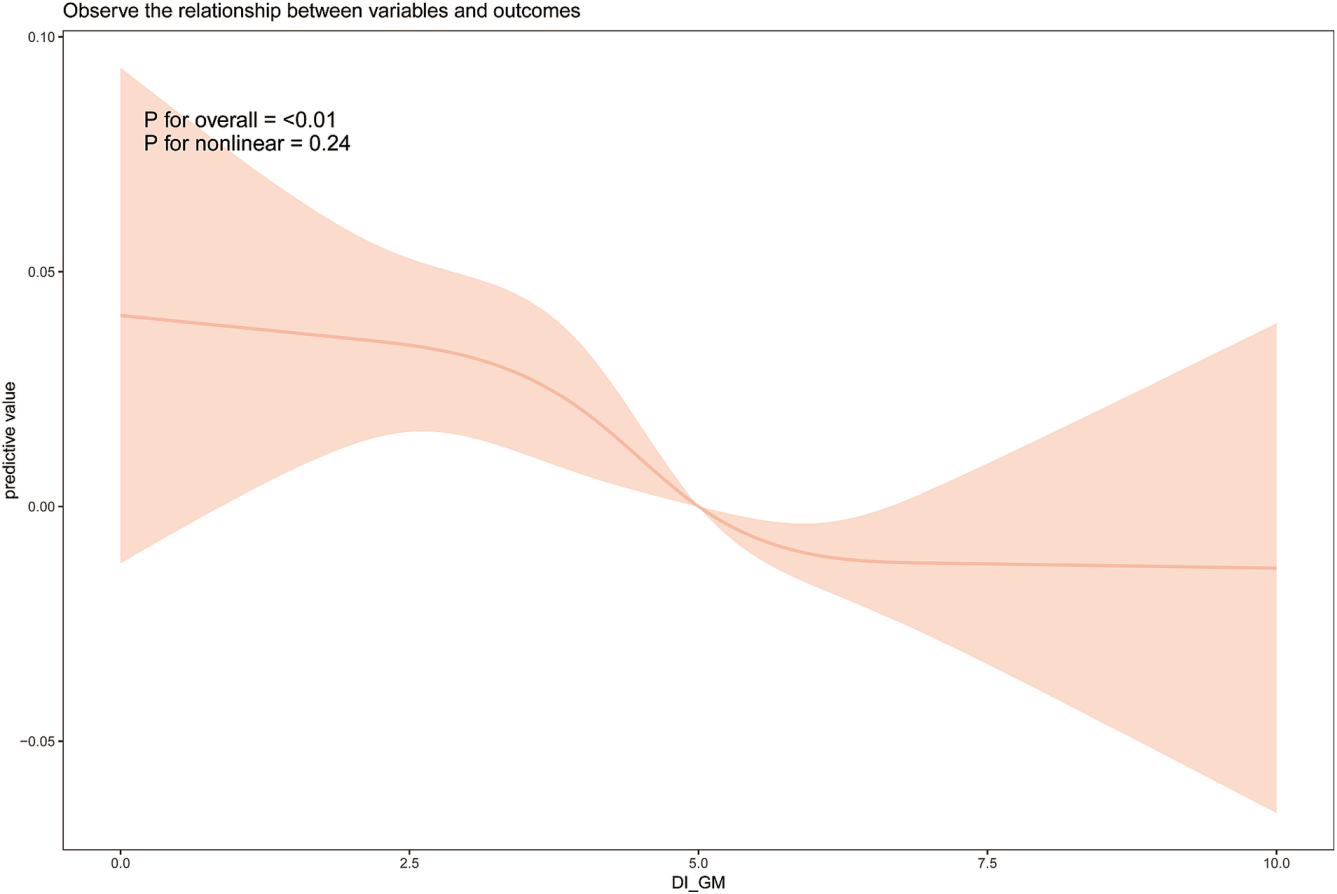
Weighted restricted cubic spline analysis of the relationship between dietary index for gut microbiota (DI-GM) and frailty. Analyses were adjusted for the variables of age, gender, race, education level, marital status, PIR, smoking, and alcohol consumption.

We further explored the relationship between DI-GM and frailty using weighted restricted cubic spline fitting and threshold effect analysis. The results, shown in Figure 2, indicate a nearly linear relationship between DI-GM and frailty after adjusting for all covariates (non-linearity *p* = 0.24). Piecewise logistic regression revealed no significant association between DI-GM and frailty when DI-GM < 3 (OR = 0.967 [0.863, 1.084]). When DI-GM > 3, a significant negative association between DI-GM and frailty was observed (OR = 0.925 [0.894, 0.957]).

### 3.4 Subgroup analysis

**Figure 3:**
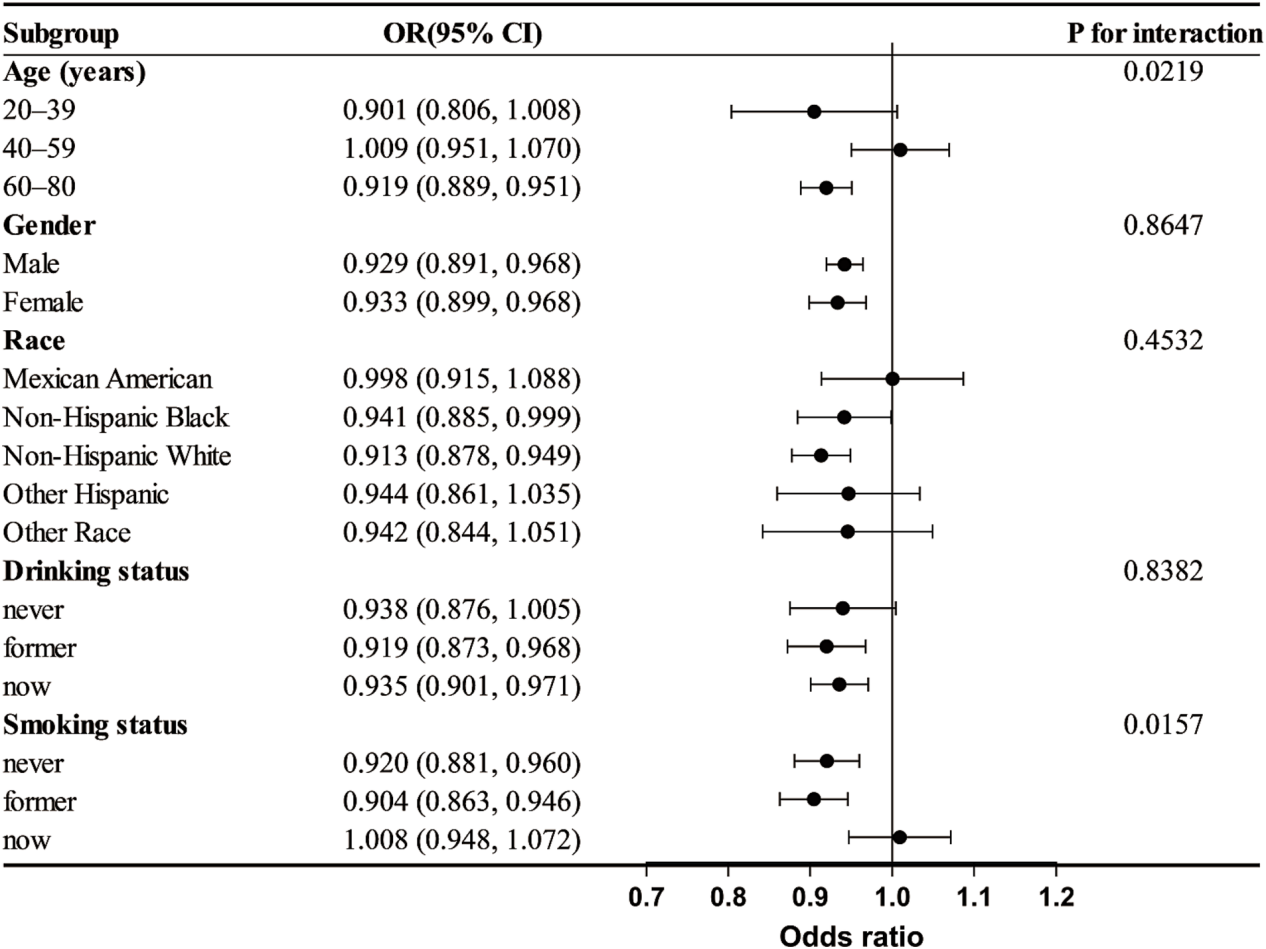
Subgroup analysis of the association between dietary index for gut microbiota (DI-GM) and frailty. Analyses were adjusted for variables such as age, gender, race, education level, marital status, PIR, smoking, and alcohol consumption.

An analysis of subgroups was performed considering age, gender, race, alcohol use, and smoking habits to investigate variations in the link between DI-GM and frailty among different subgroups. The results, shown in Figure 3, suggest that age and smoking may influence the association between DI-GM and frailty (*P* for interaction < 0.05). In individuals under 60 years old, no significant association between DI-GM and frailty was observed. However, in the ≥60 years subgroup, a significant negative association between DI-GM and frailty was found (OR = 0.919 [0.889, 0.951]). In both non-smokers and former smokers, a significant negative association between DI-GM and frailty was observed, whereas no statistically significant association was found in current smokers (OR = 1.008 [0.948, 1.072]). No significant interaction effects were found in the gender, race, and alcohol consumption subgroups (*P* for interaction > 0.05).

### 3.5 Association of albumin and HDL with DI-GM and frailty

Table 3 shows the association between DI-GM, albumin, and HDL after multivariable weighted logistic regression. In the fully adjusted model (Model 3), DI-GM was positively associated with albumin (*β* = 0.017 [0.012, 0.022]) and HDL (*β* = 0.476 [0.197, 0.755]). Table 3 also displays the association between albumin, HDL, and frailty after multivariable weighted logistic regression. The results showed that in the fully adjusted model (Model 3), both albumin (OR = 0.291 [0.238, 0.357]) and HDL (OR = 0.980 [0.975, 0.984]) were negatively associated with frailty.

**Table 3:** The associations of albumin and HDL with DI-GM and frailty.

Characteristics	DI-GM			Frailty		
	*β*	95% CI	*p*-value	OR	95% CI	*p*-value
Albumin						
Model 1	0.016	0.011, 0.022	<0.001	0.232	0.188, 0.286	<0.001
Model 2	0.019	0.014, 0.024	<0.001	0.264	0.213, 0.326	<0.001
Model 3	0.017	0.012, 0.022	<0.001	0.291	0.238, 0.357	<0.001
HDL						
Model 1	0.964	0.627, 1.300	<0.001	0.983	0.979, 0.987	<0.001
Model 2	0.691	0.377, 1.004	<0.001	0.974	0.969, 0.978	<0.001
Model 3	0.476	0.197, 0.755	0.001	0.980	0.975, 0.984	<0.001

OR, odds ratio; 95% CI, 95% confidence interval. DI-GM, dietary index for gut microbiota; HDL, high-density lipoprotein; Model 1: no adjustment for covariates. Model 2: adjusted for age, gender, and race. Model 3: adjusted for age, gender, race, education level, marital status, PIR, smoking, alcohol consumption. *P* < 0.05 is considered statistically significant.

### 3.6 Mediation effects of albumin and HDL

**Figure 4:**
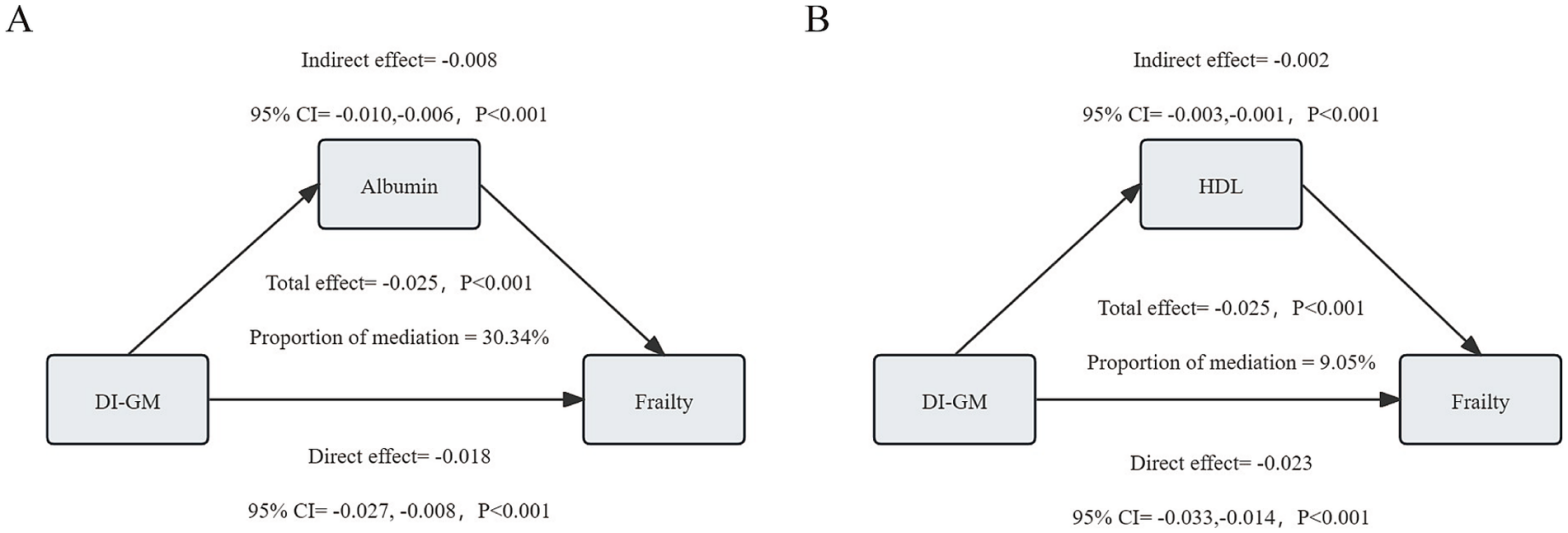
Analysis of the mediating role of albumin **(A)** and high-density lipoprotein (HDL) **(B)** on the relationship between dietary index for gut microbiota (DI-GM) and frailty.

We conducted mediation analysis to explore potential mediating mechanisms of DI-GM’s effect on frailty. As shown in Figure 4, albumin and HDL played significant mediating roles in the relationship between DI-GM and frailty. Specifically, the total effect of DI-GM on frailty mediated by albumin was-0.025 (−0.035, −0.016), with a mediation effect of-0.008 (−0.010, −0.006), accounting for 30.34% of the total effect. The total effect of DI-GM on frailty mediated by HDL was-0.025 (−0.036, −0.016), with a mediation effect of-0.002 (−0.003, −0.001), accounting for 9.05% of the total effect.

### 3.7 Dietary pattern differences across frailty statuses and DI-GM quartiles

To enhance the practical interpretability of the findings, we further analyzed dietary intake differences across frailty statuses and DI-GM score quartiles (Supplementary Figure S1). The results showed that compared to the frail group, non-frail participants consumed significantly more gut-friendly foods, including dietary fiber, fermented dairy products, soy-based foods, whole grains, broccoli, and avocado. Additionally, they had a significantly higher intake of total fat and refined grains. With increasing DI-GM scores, the intake of most beneficial dietary components showed an upward trend. In particular, participants in the Q4 group (highest DI-GM) consumed significantly higher amounts of avocado, broccoli, chickpeas, coffee, cranberry, fermented dairy products, green tea, soy-based foods, whole grains, and dietary fiber compared to the Q1 group. Moreover, Q4 participants had significantly lower intakes of total fat, refined grains, processed meat, and red meat, suggesting a healthier dietary pattern overall (*p* < 0.05).

## 4 Discussion

Frailty is acknowledged worldwide as a public health concern, especially in the elderly population. Research has shown that frailty is closely associated with the onset of various chronic diseases, including cardiovascular disease, diabetes, and cognitive dysfunction (22–24). Additionally, frailty is seen as a standalone risk factor for higher mortality rates in the elderly, necessitating urgent development of effective intervention strategies (25). This study aims to explore the relationship between DI-GM and frailty and further analyze potential mechanisms. In this study, which involved 11,163 adult participants from the NHANES database, we observed a negative correlation between DI-GM and frailty. RCS analysis showed an almost linear connection between DI-GM and frailty, with the negative correlation becoming more significant when DI-GM was at least 3. Age and smoking may influence this relationship. After confirming significant associations between albumin, HDL, DI-GM, and frailty, mediation analysis revealed the key roles of albumin and HDL in this relationship. Nutrition may be the underlying mechanism behind the association between DI-GM and frailty.

The gut microbiota plays a crucial role in regulating the immune system, and its dysregulation may contribute to the development of frailty. Studies have shown that gut microbes regulate immune tolerance and inflammation levels by modulating the host’s immune response. During early life stages, normal colonization of the gut microbiota promotes the development of gut-associated lymphoid tissue, a process that is crucial for the formation of the early immune system (26). Additionally, gut microbiota produce SCFAs, such as acetate, propionate, and butyrate, which not only enhance gut barrier and immune function, reducing the transfer of endogenous bacterial components, but also lower insulin resistance, reduce oxidative stress, and maintain central nervous system homeostasis, positively impacting diseases like stroke and Alzheimer’s disease (27). Inflammatory response is one of the key mechanisms underlying frailty. Previous studies have shown that inflammatory biomarkers and derived inflammatory indices in the blood are significantly positively correlated with frailty, indicating that frail individuals may experience a persistent inflammatory state, with gut microbiota playing a key role in this process (28). The human gut hosts millions of microbial species, which together maintain a complex balanced system that ensures proper gut function and overall health. An increase in pro-inflammatory microbiota and a decrease in anti-inflammatory microbiota leads to an inflammatory gut environment, which damages the gut barrier, promotes the entry of endotoxins like lipopolysaccharides into the bloodstream, and triggers systemic inflammation (29). Persistent inflammatory states impair immune function, accelerate muscle atrophy, and decrease physical capacity, ultimately leading to the onset or worsening of frailty (30, 31).

The gut microbiota plays a crucial role in nutrient absorption, and nutritional deficiencies are one of the primary manifestations of frailty. Gut microbiota directly affect the host’s nutritional status by breaking down dietary components and synthesizing vitamins and other essential nutrients (32). Species such as Bifidobacterium, Firmicutes, and Enterococcus ferment carbohydrates in the colon, synthesizing SCFAs. In addition to providing direct energy to the body, SCFAs promote the health of intestinal epithelial cells and feedback to enhance the gut’s ability to absorb nutrients (33). Multiple mouse studies have shown that gut microbiota play a key role in promoting lipid metabolism, significantly enhancing the metabolic efficiency of triglycerides, cholesterol, and other lipids (34). Additionally, gut microbiota produce specific proteases, such as histidine decarboxylase and glutamate decarboxylase, further promoting protein degradation and enhancing its bioavailability (35, 36).

The homeostasis of the gut microbiota is influenced by multiple factors, including age, genetics, and lifestyle, with dietary factors, in particular, gaining increasing attention from both the scientific community and the public in recent years (37, 38). Research has demonstrated that diet is a primary factor in altering the composition of the gut microbiota. A “Western diet” rich in high-fat and high-sugar foods leads to a reduction in Bacteroidetes and an increase in Firmicutes, while populations consuming a Mediterranean diet rich in fiber and omega-3 fatty acids show higher levels of Bacteroidetes and Bifidobacterium in their gut microbiota (39). Kase et al. (14), through a systematic analysis of extensive literature, developed the DI-GM index based on 14 foods and nutrients associated with the gut microbiota, which systematically quantifies the impact of individual diet on the gut microbiota. We found a negative correlation between the DI-GM index and frailty, highlighting the significant potential of dietary factors in combating frailty.

We found that the DI-GM index was approximately linearly associated with frailty, with each one-unit increase in DI-GM score associated with an approximately 6% reduction in frailty risk. Considering the high prevalence of frailty among older adults, even a modest risk reduction may lead to substantial health benefits at the population level, making dietary interventions particularly valuable due to their low cost and high feasibility. The dose–response relationship shows a more significant negative correlation with frailty when the DI-GM index exceeds 3. This finding emphasizes the importance of a rich dietary composition, where dietary diversity and overall nutritional completeness play a more critical role in health regulation than the quantity of a specific food component. Additionally, subgroup analysis revealed a more significant negative correlation between the DI-GM index and frailty in older adults, whereas this association was weaker in current smokers. Smoking is known to have long-term negative effects on the gut microbiota. Smoking alters the composition of the gut microbiota, suppressing the growth of beneficial bacteria while promoting the proliferation of harmful bacteria (40). Ribière et al. (41) noted that smoking also damages gut barrier function and increases intestinal inflammation, factors that may interfere with the beneficial regulatory effects of dietary components on the gut microbiota. Although dietary improvements can enhance gut health, in smokers, the imbalance in the gut microbiota may weaken the beneficial effects of diet on frailty due to the adverse impacts of smoking. In contrast, older adults may be more sensitive to dietary changes. As individuals age, their immune function and gut health gradually decline. Dietary diversity and beneficial components effectively promote gut microbiota balance, improve immune function, reduce inflammation, and thus decrease the incidence of frailty (38, 42).

In our study, albumin and HDL were found to be significantly associated with both the DI-GM index and frailty. Further mediation analysis revealed the key role of these two biomarkers in the relationship between DI-GM and frailty. Albumin, the major plasma protein synthesized by the liver, is closely related to protein synthesis, immune function, and anti-inflammatory responses. HDL, widely regarded as a protective lipoprotein, possesses antioxidant, anti-inflammatory, and cholesterol reverse transport functions (43–45). The mediation analysis revealed the roles of these biomarkers, offering new insights into the complex biological mechanisms underlying the relationship between DI-GM and frailty. The DI-GM diet not only directly affects the gut microbiota but may also indirectly reduce the risk of frailty by improving albumin and HDL levels.

Our findings suggest that the DI-GM index could serve as a community-level nutritional screening tool to help identify individuals with suboptimal dietary patterns at an early stage. Clinically, the DI-GM may serve as a novel indicator for evaluating responses to dietary interventions, facilitating the development of an integrated “diet–microbiota–frailty” assessment framework. Informed by previous studies, we propose several practical dietary recommendations for healthcare professionals, including: (1) consume at least 25 grams of dietary fiber per day from sources such as legumes, whole grains, and dark green vegetables; (2) increase the intake of fermented dairy products; and (3) consume green tea in moderation and reduce the intake of processed meats and refined grains (46, 47). As a quantitative index of diet–microbiota interaction, the DI-GM holds promise for optimizing existing dietary guidelines, particularly for older adults with frailty or at nutritional risk, and may help advance precision nutrition strategies.

This study has several advantages. We conducted a weighted analysis of participants from NHANES 2007–2018, ensuring that the results are representative and reliable. Furthermore, we are the first to demonstrate the association between DI-GM and frailty, showing that improving the dietary quality of older adults can effectively reduce the risk of frailty, offering new perspectives for elderly health management. By promoting the application of the DI-GM score, clinicians can better identify high-risk individuals and develop targeted interventions.

This study has several limitations. First, dietary data were self-reported by participants, which may be subject to recall bias, daily variability, and subjective interpretation, potentially affecting the accuracy of DI-GM assessment. Second, although multiple confounding variables were controlled for, unmeasured or residual confounding may still exist, potentially affecting the robustness of the findings. In addition, the study sample was primarily drawn from the U.S. population, whose sociocultural background and dietary habits may limit the generalizability of the findings to other countries and regions. Finally, due to the cross-sectional design of the study, causal relationships and temporal sequencing among DI-GM (exposure), albumin and HDL (mediators), and frailty (outcome) could not be established. Mediation analysis is generally grounded in a causal inference framework that assumes a temporal order among variables, which could not be fully satisfied in the current study. Therefore, the findings should be interpreted with caution. Future studies are recommended to employ longitudinal designs or randomized controlled trials to further clarify the potential causal mechanisms.

## 5 Conclusion

In conclusion, this study reveals a significant negative correlation between DI-GM and frailty, suggesting that improving dietary patterns may be an effective strategy to reduce frailty risk. These findings may provide a basis for the formulation of public health policies. Future research should further explore the clinical applications of DI-GM and its role in health management, aiming to provide a stronger scientific foundation for clinical practice.

## Data Availability

Publicly available datasets were analyzed in this study. This data can be found: https://www.cdc.gov/nchs/nhanes/index.htm.
