# Supplementary material for: The association between the dietary index for gut microbiota and frailty: a cross-sectional study
Source: Front Nutr. 2025 May 1;12:1580753. doi: 10.3389/fnut.2025.1580753 (PMC12080234; doi:10.3389/fnut.2025.1580753)
Supplement: Supplementary file 1 [file Table_1.DOCX]

Supplementary Material

**Supplementary Table 1.** Components and scoring criteria of DI-GM.

| Components of DI-GM | Food and nutrients. | Scoring standards |
| --- | --- | --- |
| Beneficial to gut microbiota | Avocados | For each component that benefits the gut microbiota, a score of 1 is assigned if the intake is equal to or greater than the sex-specific median; otherwise, the score is 0. |
|  | Broccoli |  |
|  | Chickpeas |  |
|  | Coffee |  |
|  | Cranberries |  |
|  | Fermented dairy (including yogurt, cheese, kefir, sour cream, buttermilk) |  |
|  | Fiber |  |
|  | Green tea |  |
|  | Soybean (including Soy milk, Tofu) |  |
|  | Whole grains (grains defined as whole grains, containing the entire grain kernel—the bran, germ, and endosperm) |  |
| Harmful to the gut microbiota. | High-fat diet (% energy) | A score of 0 is given if the intake is at or above 40% of total energy from fat; otherwise, the score is 1.For each other component that is detrimental to the gut microbiota, a score of 0 is assigned if the intake is equal to or greater than the sex-specific median; otherwise, the score is 1. |
|  | Processed meat (including frankfurters, sausages, corned beef, and luncheon meat that are made from beef, pork, or poultry) |  |
|  | Red meat (including beef, veal, pork, lamb, and game meat; excludes organ meat and cured meat) |  |
|  | Refined grains (refined grains that do not contain all of the components of the entire grain kernel) |  |

DI-GM,Dietary index for gut microbiota.

**Supplementary Table 2.** Components and scoring criteria of FI.

| Anthropometric data | BMI | <18.5 or ≥30 = 1 |
| --- | --- | --- |
|  |  | 18.5 to 25 = 0 |
|  |  | 25 to 30 = 0.5 |
|  | Handgrip strength | Male:  For BMI 24 to 28, GS ≤ 30 = 1;  For BMI ≤ 24, GS ≤ 29 = 1;  For BMI >28, GS ≤ 32 = 1.  Female:  For BMI 23 to 26, GS ≤17.3 = 1;  For BMI>29, GS ≤ 21 = 1.  For BMI ≤23, GS ≤17 = 1;  For BMI 26 to 29, GS ≤ 18 = 1; |
| Chronic disease comorbidity | Chronic bronchitis | No = 0  Suspect = 0.5  Yes = 1 |
|  | Thyroid problems |  |
|  | Arthritis |  |
|  | Congestive heart failure |  |
|  | Cancer |  |
|  | Coronary heart disease |  |
|  | Angina |  |
|  | Weak/failing kidneys |  |
|  | Heart attack |  |
|  | Blood pressure |  |
|  | Stroke |  |
|  | Urinary Leakage |  |
|  | Diabetes |  |
|  | Experience confusion/memory problems | Yes = 1  No = 0 |
| Hospital Utilization and Access to Care | Number of prescribed medications | None = 0, 1 to 4 = 0.5, ≥5 = 1 |
|  | Overnight hospital patient in past year | Yes = 1  No = 0 |
|  | Health now compared with 1 year ago | Worse = 1  About the same, Better = 0 |
|  | Self-rated health | Fair or poor = 1  Excellent, very good, or good = 0 |
|  | Frequency of healthcare use during the past year | None = 0, 1 to 5 = 0.5, More than 5 = 1 |
| Physical dependency | Managing money | Difficulty = 1  No Difficulty = 0 |
|  | Stooping, crouching, kneeling |  |
|  | Walking up 10 steps difficulty |  |
|  | Lifting or carrying |  |
|  | Walking for a quarter mile difficulty |  |
|  | House chore |  |
|  | Attending social events |  |
|  | Preparing meals |  |
|  | Standing for long periods difficulty |  |
|  | Standing up from armless chair |  |
|  | Dressing yourself |  |
|  | Using fork, knife, drinking from cup |  |
|  | Grasp/holding small objects |  |
|  | Getting in and out of bed difficulty |  |
|  | Push or pull large objects |  |
| Laboratory Values | Glycohemoglobin (%) | 0% to 5.7% = 0, >5.7% = 1 |
|  | Segmented neutrophils percent (%) | 40 to 80 = 0, Other = 1 |
|  | Lymphocyte percent (%) | 20 to 40 = 0, Other = 1 |
|  | Red cell distribution width (%) | 11.6 to 14.6 = 0, Other = 1 |
|  | Hemoglobin (g/dL) | Male: 13.5 to 18 = 0, Other = 1  Female: 12 to 16 = 0, Other = 1 |
|  | Red blood cell count (million cells/ml) | Female: 4.2 to 5.4 = 0, Other = 1  Male: 4.7 to 6.1 = 0, Other = 1 |
| Depressive Symptoms | Trouble sleeping or sleeping too much | Several days = 0.33  Nearly every day = 1  Not at all = 0  More than half the days = 0.66 |
|  | Trouble concentrating on things |  |
|  | Have little interest in doing things |  |
|  | Feeling tired or having little energy |  |
|  | Feeling down, depressed, or hopeless |  |
|  | Poor appetite or overeating |  |
|  | Feeling bad about yourself |  |

FI,Frailty index;BMI,Body mass index

**Supplementary Table 3.** Analysis of the mediation by albumin and HDL of the associations of DI-GM with frailty.

| Characteristics | Mediation effect (95% CI), P value | | |  |
| --- | --- | --- | --- | --- |
|  | Total effect | Indirect effect | Direct effect | Mediation |
| Albumin | -0.025(-0.035,-0.016) <0.001 | -0.008(-0.010,-0.006) <0.001 | -0.018 (-0.027, -0.008) <0.001 | 30.34% |
| HDL | -0.025(-0.036,-0.016) <0.001 | -0.002(-0.003,-0.001) <0.001 | -0.023(-0.033,-0.014)  <0.001 | 9.05% |

HDL, high-density lipoprotein; Adjusted for age, gender, race, education level, marital status, PIR, smoking, alcohol consumption. P < 0.05 is considered statistically significant.


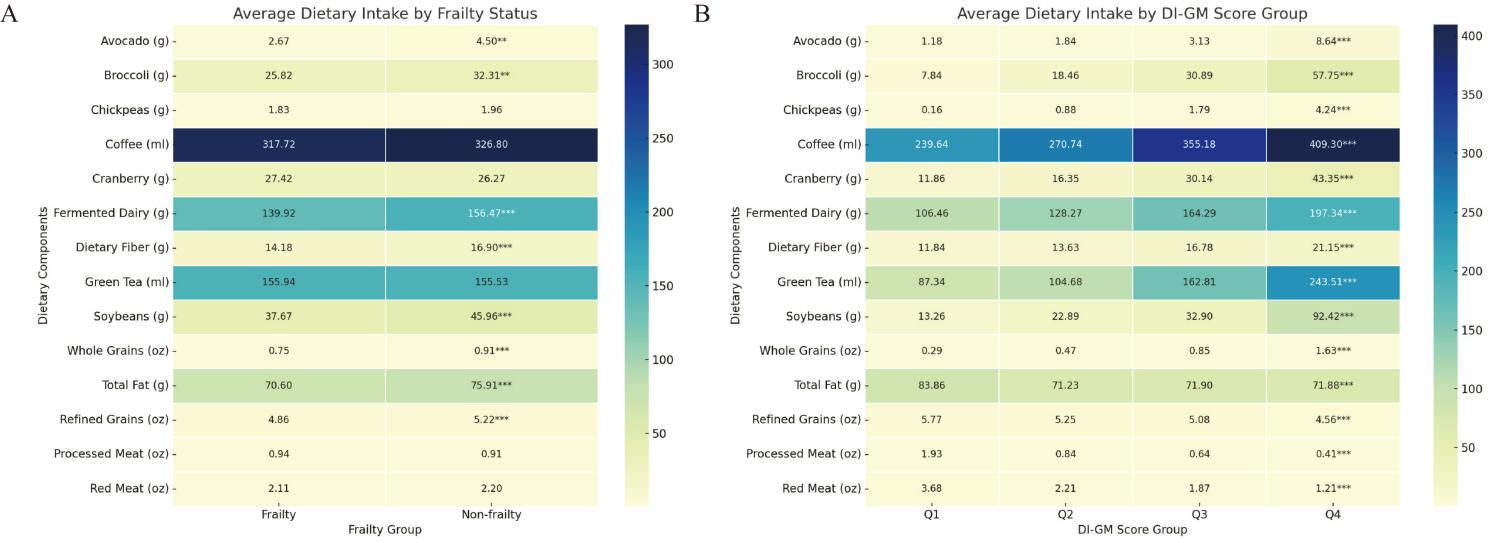


**Supplementary Figure 1.**Average daily intake of gut microbiota(A) Mean intake of selected dietary components among participants classified as frail vs. non-frail. (B) Mean intake by DI-GM score quartiles (Q1–Q4), where Q4 represents the highest gut microbiota–friendly dietary index. Asterisks indicate statistically significant differences between Q1 and Q4 groups.Values are presented to two decimal places.Significance levels: *p < 0.05; **p < 0.01; ***p < 0.001.
